# Oxidative Stress-Related Genetic Variants May Modify Associations of Phthalate Exposures with Asthma

**DOI:** 10.3390/ijerph14020162

**Published:** 2017-02-08

**Authors:** I-Jen Wang, Wilfried J. J. Karmaus

**Affiliations:** 1 Department of Pediatrics, Taipei Hospital, Ministry of Health and Welfare, Taipei 11267, Taiwan; 2 Institute of Environmental & Occupational Health Sciences, School of Medicine, National Yang-Ming University, Taipei 100044, Taiwan; 3 Department of Health Risk Management, China Medical University, Taichung 110001, Taiwan; 4 Division of Epidemiology, Biostatistics, and Environmental Health, School of Public Health, University of Memphis, Memphis, TN 38152, USA; karmaus1@memphis.edu

**Keywords:** phthalate, asthma, oxidative stress, *SOD2*, *GSTP1*

## Abstract

*Background:* Phthalate exposure may increase the risk of asthma. Little is known about whether oxidative-stress related genes may alter this association. First, this motivated us to investigate whether genetic polymorphisms of the oxidative-stress related genes glutathione *S*-transferase Mu 1 (*GSTM1*), glutathione *S*-transferase pi 1 (*GSTP1*), superoxide dismutase 2 (*SOD2*), catalase (*CAT*), myeloperoxidase (*MPO*), and *EPHX1* in children are associated with phthalate urine concentrations. Second, we addressed the question whether these genes may affect the influence of phthalates on asthma. *Methods:* In a case-control study composed of 126 asthmatic children and 327 controls, urine phthalate metabolites (monoethyl phthalate (MEP), monobutyl phthalate (MBP), monobenzyl phthalate (MBzP), and mono(2-ethyl-5-hydroxyhexyl)phthalate (MEHHP) were measured by UPLC-MS/MS at age 3. Genetic variants were analyzed by TaqMan assay. Information on asthma and environmental exposures was also collected. Analyses of variance and logistic regressions were performed. *Results:* Urine MEHHP levels were associated with asthma (adjusted OR 1.33, 95% CI (1.11–1.60). Children with the *GSTP1* (rs1695) AA and *SOD2* (rs5746136) TT genotypes had higher MEHHP levels as compared to GG and CC types, respectively. Since only *SOD2* TT genotype was significantly associated with asthma (adjusted OR (95% CI): 2.78 (1.54–5.02)), we estimated whether *SOD2* variants modify the association of MEHHP levels and asthma. As MEHHP concentrations were dependent on *GSTP1* and *SOD2*, but the assessment of interaction requires independent variables, we estimated MEHHP residuals and assessed their interaction, showing that the OR for *SOD2* TT was further elevated to 3.32 (1.75–6.32) when the residuals of MEHHP were high. *Conclusions:* Urine phthalate metabolite concentrations are associated with oxidative-stress related genetic variants. Genetic variants of *SOD2*, considered to be reflect oxidative stress metabolisms, might modify the association of phthalate exposure with asthma.

## 1. Introduction

Human exposure to phthalates mainly occurs through ingestion, inhalation and dermal absorption [1]. In addition to significant changes of immunological and inflammatory biomarkers (total IgE, IL-4, IFN-γ and eosinophils), pulmonary histological and physiological data support the idea that di(2-ethylhexyl) phthalate (DEHP) may promote and aggravate allergic asthma [2]. In Sweden, a case-control study found that the risk of asthma in children was related to the concentration of DEHP in house dust [3]. In Bulgaria, the concentration of DEHP in indoor dust was related to wheezing among preschool children in a case-control study [4]. In the USA, urinary phthalate metabolites were positively associated with airway inflammation in an urban ongoing birth cohort [5]. Repeated exposure to airborne DEHP has also been shown to increase the levels of inflammatory cells, including eosinophils, lymphocytes, and neutrophils, in the lung and bronchoalveolar lavage fluid [6]. Another study showed that exposure to high concentration of airborne mono(2-ethyl-5-hydroxyhexyl)phthalate (MEHHP) resulted in acute airway irritation and an increased number of macrophages in the bronchoalveolar lavage fluid [7]. These findings suggest that phthalate exposure early in life may constitute an increased susceptibility to asthma development and an association observed in existing asthma. Asthma is a complex pulmonary inflammatory disease, which is characterized by airway hyperresponsiveness, variable airflow obstruction and inflammation in the airways. Children with asthma often suffer from considerable school absences, family stress, and health care expenditures [8]. However, effects of phthalate exposure on the development of allergic diseases remains unclear. In a prior study, we found that urine phthalate metabolites were associated with the skin-related filaggrin gene and atopic dermatitis, suggesting a higher skin absorption [9]. In this study, we focus on effects of urine phthalate metabolites on asthma and tested whether genetic polymorphisms of oxidative-stress related genes in concert with phthalate metabolites affect the airways. It has been reported that an adjuvant effect induced by DEHP is mediated through oxidative stress in a mouse model of asthma [10]. Oxidative stress may originate from reactive oxygen and nitrogen species generated as a consequence of activated inflammatory and bronchial epithelial cells after tissue injury following the inhalation of chemicals [11]. In a mouse model, expression of genes involved in oxidative stress and thiol redox balance was increased when mice were exposed to polymeric hexamethylene diisocyanate [12]. Moreover, an imbalance in redox systems favoring an oxidative environment has been associated with airflow limitation and airway remodeling in asthma [13]. A number of enzymatic antioxidants, including glutathione *S*-transferases (*GSTs*), superoxide dismutase 2 (*SOD2*), microsomal epoxide hydrolase (*EPHX1*), catalase (*CAT*), and myeloperoxidase (*MPO*) play a role in such a redox imbalance in the lung and may initiated inflammatory responses [14,15]. In addition, these five gene have also been associated with the occurrence of asthma in different exposure settings and oxidative stress-related genetic variants of *CAT*, *SOD2* and *MPO* have been reported to modify the association of urine phthalate metabolites levels and pulmonary function [16,17,18,19]. Following these considerations, we hypothesize that genetic variants considered to be involved in oxidative stress pathways modify the association of phthalate with asthma.

Given that children are more susceptible to environmental exposures and that oxidative stress may be an influential mechanism, further investigations into the effect of environmental phthalate exposure on asthma and identifying possible biological explanation are important. This motivated us to investigate in step 1 whether specific genetic polymorphisms of oxidative stress markers, glutathione *S*-transferase Mu 1 (*GSTM1*), glutathione *S*-transferase pi 1 (*GSTP1*), *SOD2*, *CAT*, *MPO*, and *EPHX1* are related to a higher urine concentration of phthalates and then in step 2 whether these genes may modify effects of phthalates on asthma.

## 2. Methods

### 2.1. Study Population

We conducted a case-control study comprised of 126 asthma children and 327 controls. Three control subjects were matched to case subjects by date of enrollment (within 3 months) if both had urine specimens and oral scrapes. All children were part of the Childhood Environment and Allergic diseases Study (CEAS) cohort recruited in 2010 in Taipei [20]. At the age 3, phthalate metabolites were measured in urine samples, and genotyping was based on buccal cells samples. Parents were interviewed using a standardized questionnaire regarding basic demographics, birth history, parental age and education levels, family income, parental history of allergic diseases, duration of breast feeding, and environmental exposures of the child. Information about asthma was also collected. Written informed consent was obtained from all parents. The study protocol was approved by Taipei hospital’s Institutional Review Board (TH-IRB-11-02).

### 2.2. Case Definition and Assessment of Confounders

The International Study of Asthma and Allergies in Childhood (ISAAC) questionnaire was applied and post-natal exposures such as furry pets, carpets, or incense burning at home, fungi at house walls, and environmental tobacco smoke exposure were evaluated. Regarding the latter, we ask the parents “In the past year, has your child been exposed to environmental tobacco smoke? How many hours per day was he/she exposed”? At the clinics, experienced pediatric allergists performed a standardized history and clinical examination of participants with a parental-reported doctor-diagnosed asthma from the ISAAC questionnaire. Cases with asthma were confirmed by pediatric allergists based on the following three criteria according to the GINA Guidelines 2015: (i) recurrence of at least two of the three symptoms: cough, wheeze, and shortness of breath within the previous 12 months without having a cold; (ii) doctor’s diagnosis of asthma with ongoing treatment; (iii) response to treatment with β2-agonists or inhaled corticosteroids [21].

### 2.3. Laboratory Methods

#### 2.3.1. Exposure Monitoring

First mid-stream 10 mL urine in the morning were collected into glass containers and stored at −20 °C until analysis. Four phthalate metabolites (monoethyl phthalate (MEP), monobutyl phthalate (MBP), monobenzyl phthalate (MBzP), and mono(2-ethyl-5-hydroxyhexyl)phthalate (MEHHP)) representing the exposure to four commonly used phthalates (diethylphthalate (DEP), dibutyl phthalate (DBP), butyl benzyl phthalate (BBzP), and di(2-ethylhexyl) phthalate (DEHP)) were measured by ultra-performance liquid chromatography coupled with tandem mass spectrometry (UPLC-MS/MS) (Waters, Milford, MA, USA), as described previously [22]. Details are provided in the Supplementary Material (last page). The limit of detection for MEP, MBP, MBzP, MEHHP were 3.27, 0.95, 0.15, and 1.36 ng/mL. For concentrations below the detection limits, a value of half the limit of detection was assigned. All concentrations are based on duplicate analysis. Regarding the procedure for avoiding contamination, the adsorbent was washed with twice of 0.1% NH_4_OH in methanol and twice of Milli-Q water. All glassware was rinsed with acetone and methanol sequentially after washing and before the use. All adsorbent plate was disposed after the extraction. Each batch of samples contained a reagent blank, matrix blank, two matrix spike samples, sample duplicate, and sample spike. No contamination of analytes was identified. Urine creatinine levels were analyzed by enzymatic assay according to the manufacturers’ instructions (Cayman Chemical, Ann Arbor, MI, USA) [23]. All statistical models were adjusted for urine creatinine levels.

#### 2.3.2. Genotyping

Genomic DNA was extracted from buccal cells by Gentra Purgene DNA Buccal Cell Kit (QIAGEN Sciences, Germantown, MD, USA). Candidate genes were selected based on their functional role in oxidative stress and inflammation. The QuickSNP version 1.1 (Carlsbad, CA, USA) was used to select 9 tag single nucleotide polymorphism (SNPs) within the candidate genes that had a minor allele frequency (MAF) > 5% and an r2 > 0.8 in Asian populations [24]. The *GSTM1* (null or non-null genotype), *GSTP1* (rs1695), *SOD2* (rs5746136), *SOD2* (rs4880), *CAT* (rs769218), *MPO* (rs2071409), *EPHX1* (rs1051740), and *EPHX1* (rs2740171) polymorphisms (Table S1), selected from hot spots of Asian populations on NCBI website, were analyzed by TaqMan SNP Genotyping Assay (ABI, Foster City, CA, USA) as described elsewhere [25]. In addition, deletion of *GSTM1* genes were analyzed. To control for errors and technical problems in genotyping, derived genotype frequencies were compared with the expected allelic population equilibrium based on the Hardy-Weinberg equilibrium test. Incomplete data missing at least one of two SNPs were excluded, and then individual haplotypes were estimated from genotype data.

### 2.4. Statistical Analysis

Baseline characteristics were compared between case and control subjects with two-sample *t*-test for continuous variables or Chi-square tests for categorical variables. Skewed data were log (Ln)-transformed before further analyses. All log-transformed data showed a normal distribution; no influential outliers were found. Differences of geometric means of phthalate metabolite levels among different genotypes were determined by analysis of covariance. For the association of genetic variants with asthma, we estimated odds ratios (ORs) and their 95% confidence intervals (95% CIs) using logistic regression analysis. Then, we determined whether one of the oxidative stress genes had an effect on the distribution of the four phthalate markers. Since some phthalate metabolite concentration seems to depend on oxidative stress genotypes, we were not allowed to simply test the interaction of phthalate with the oxidative stress genotypes, as unbiased assessments of interaction need to be based on two independent variables [26]. To remove the dependency, we first regressed the phthalate markers on the oxidative stress genotypes and estimated the residuals of the phthalate concentration that is not explained by oxidative stress genotypes. Then we tested the interaction of the phthalate residuals with the oxidative stress genes on asthma. The interactive effect was estimated only in the presence of statistically significant interaction term (*P*_interaction_ ≤ 0.05) as the following:

OR = exp(β_1_ × covariate_1_ + β_2_ × covariate_2_ + β_3_ × (covariate_1_ × covariate_2_)) (1)

Bonferroni correction was used to address the problem of multiple comparisons. Potential confounders identified in the literature such as gender, premature birth, maternal age and education, maternal history of atopy, family income, duration of breast feeding, number of older siblings, pet raising, environmental tobacco exposure (ETS), incense burning, carpets at home, and fungi on house walls were taken into consideration. We adjusted for those confounder that resulted in a 10% change in point estimates when removing from the model. All hypothesis testing was two-sided at a significance level of 0.05 and was performed with the SAS software version 9.1 (SAS Institute Inc., Cary, NC, USA).

## 3. Results

The characteristics of the cases and controls are outlined in Table 1. There are no significant differences between cases and controls with the exception of maternal history of atopy and environmental tobacco smoke (ETS) exposure. The distribution of urine phthalate metabolite levels was shown in Table S2.

### 3.1. Phthalate Metabolite Levels and Asthma

MEHHP levels with median, 25% tile, and 75% tile were 4.14, 4.14, and 12.12 ng/mL. MEHHP levels were significantly associated with asthma (adjusted OR 1.33, 95% CI (1.11–1.60)) (*p* = 0.008). Other urine phthalate metabolites are also positively associated with asthma, but failed to reach statistical significance (Table 2 and Table S3).

### 3.2. The Association of Different Genotypes and Asthma

A significant different effect of genotypes was found among asthma cases and controls for *GSTP1* (rs1695) and *SOD2* (rs5746136) polymorphisms; however, only the effect of *SOD2* survived the penalty for multiple testing (Table 3).

The *SOD2* (rs5746136) was significantly associated with a higher odds of asthma for the TT genotype with adjusted OR (95% CI) of 2.78 (1.54–5.02) (Bonferroni *p* = 0.009) compared with the CC genotype.

### 3.3. The Association of Phthalate Metabolite Levels and Different Genotypes

Children with the *GSTP1* AA genotype and *SOD2* TT genotype had higher MEHHP levels as compared with GG and CC carriers, respectively, adjusting for urine creatinine, age, gender, maternal history of atopy, and ETS exposure (geometric mean(se) 6.95 (3.12) vs. 3.35 (2.81), *p* = 0.011 and 7.76 (3.67) vs. 5.08 (2.76), *p* = 0.002) (Table 4). Since MEP was not significantly associated with asthma while MEHHP was associated with both *GSTP1* and *SOD2* and asthma, we focused our further analyses on the MEHHP metabolite. This strategy addressed the role of the interplay of MEHHP and the two genes for asthma.

### 3.4. The Effect of SOD2 Genotypes with MEHHP on Asthma 

Further, since *SOD2* was associated with asthma, but *GSTP1* was not (Table 3), and MEHHP urine levels differ with genotypes of *SOD2* (Table 4), we focused on the interplay of *SOD2* and MEHHP with asthma. Before we can estimate whether there is an interaction between *SOD2* and MEHHP resulting in a higher odds of having asthma, we need to estimate the part of MEHHP that is not explained by *SOD2* (the residuals). Since *SOD2* and *GSTP1* are associated (*p*-value = 0.001, Table S4), the phthalate residuals not explained by *SOD2* and *GSTP1* were estimated with both genes in the regression model. Then we tested the interaction of the phthalate residuals with *SOD2* gene on asthma. Table 5 shows that the residual of MEHHP in interaction with SOD2 statistically significantly increase the odds of asthma. The interactive effect of SOD2 TT with the residual of MEHHP can be estimated by: OR = exp(β1 × SOD2 TT + β2 × residual of MEHHP + β3 × (SOD2 TT × residual of MEHHP)) = exp(1.112 × SOD2 TT − 0.047 × residual of MEHHP + 0.055 × (SOD2 TT × residual of MEHHP)) = 3.04 (Table 5). To further demonstrate this effect in a simpler model, we stratified by the residual MEHHP levels. In addition, we controlled for known confounding factors such as urine creatinine, age, gender, maternal history of atopy, maternal education, and environmental tobacco smoke exposure. In the stratified analysis (Table 5, upper part, stratified by the limit of quantification of MEHHP), we can see that among the cases with lower MEHHP levels (≤4.14 ng/mL) there are 17.3% with the TT *SOD2* genotype, similarly among the controls (13.1%). However, among cases with higher MEHHP levels (>4.14 ng/mL), there are 35.6% with the TT *SOD2* genotype but only 15.3 in the controls. From the lower half of Table 5, addressing interaction on a multiplicative level, it is obvious that the odds. for individual with asthma is 3.04 times higher when both MEHHP residual levels are higher and the child has a TT *SOD2* genotype. Even the heterozygous *SOD2* genotype (TC) is related to a moderately increased odds, when MEHPP levels are higher. This a genetic “dose-response” relationship (CC→CT→TT), strengthening the idea that an interaction between the *SOD2* genotype and MEHHP increased the risk of asthma. 

The interaction term on a multiplicative level is statistically significant, but does not represent the total effect of *SOD2* and MEHHP, since both main effects are not included in the OR of 3.04. The additive interaction, determined by stratification, provides an idea of the public health importance (OR = 8.08).

## 4. Discussion

The results corroborate findings of others that phthalate may be a risk factor for asthma. Our findings further suggest that *GSTP1* and *SOD2* variants are associated with urine phthalate metabolites. In addition, genetic variants to *SOD2* in interaction with MEHHP seems to constitute a higher risk of asthma. To the best of our knowledge, interactions between oxidative stress genotypes and phthalate metabolite levels in children has not been reported previously. Our findings suggest a possible role of the *SOD2* gene modifying the association of phthalates with asthma.

### 4.1. The Association of Phthalate Metabolite Levels and Oxidative-Stress Related Genes

Our analyses by *GSTP1* and *SOD2* genotypes revealed that children with the *GSTP1* AA genotype and *SOD2* TT genotype had higher MEHHP levels (Table 3). Our findings raise the question how the association of oxidative stress genes with phthalate exposure can be explained. MEHHP levels could differ by genotypes since the enzymes coded by the genes may have different roles. Glutathione *S*-transferase (GST), a well-studied phase II enzyme, is involved in detoxification of both reactive tobacco metabolic intermediates and reactive oxygen species (ROS) [27]. *SOD2* has the distinction of being the only enzyme in the mitochondria that can neutralize superoxide [28]. A functional polymorphism in exon 2 of *SOD2* gene Ala16Val (rs4880) may result in decreased antioxidant potential to limited post-transcriptional transport [29]. Substitution of C to T, that is alanine to valine, resulted in structural alterations in the mitochondrial targeting domain from β-sheet to α-helix, which induces a 30%–40% increase in *SOD* activity in mitochondria [29]. All these are oxidative stress-related genes. Individuals with a protective genotype in these genes have a first line of defense against free radicals. Oxidative stress has been linked to phthalate-induced toxicity in tissues [30]. It has been reported that DEHP may inhibit follicle growth by inducing production of ROS and by decreasing the expression and activity of superoxide dismutase [31]. A significant increase in the level of ROS was observed in arrested *SOD* following MEHP treatment [32]. DEHP increased ROS generation and decreased expression of *SOD*, glutathione peroxidase (GPX), heme oxygenase (HO), and *CAT* [33]. Phthalate exposure was also reported to induce oxidative stress and immune related gene expression [34]. Since few reports were available for the other genes used in our analysis regarding associations with toxicological or epidemiological findings, additional research is needed for other mechanisms.

### 4.2. Phthalate Metabolite Levels and Asthma 

Urine MEHHP levels were higher in children with asthma than in controls. However, there was no significant relationship between other phthalate metabolites and asthma. We assumed that the difference may be due to the different metabolizing processes that the short- and long-chain branched phthalates undergo. It is documented that MEHHP and MEOHP, metabolites from long-chain branched phthalates DEHP, continue further hydroxylation and oxidation, while short-chain branched phthalates such as MBP do not [35,36]. Levels of MBzP in Taiwanese children were much lower than the U.S. and German children [37,38]. Since other epidemiological studies point to an association between DEHP exposure and asthma in children [3,4], it is possible that higher phthalate metabolite levels in children with asthma are considered to be due to poor metabolism of reactive oxygen species in children with *GSTP1* AA genotype and *SOD2* TT genotype [19]. This would explain the gene × phthalate interaction; however, not the main effect of *SOD2*. Although various metabolizing processes may contribute to the difference, another important reason may be that children with asthma had higher environmental exposure to phthalates, if children with asthma spent more time indoors. For example, indoor exposure pathways have been reported to contribute substantially to the urinary levels of phthalate metabolites [1]. Hence, preventive measures for environmental phthalate exposure are important.

### 4.3. The Association of Oxidative-Stress Related Genes and Asthma

Our results support the involvement of genetic polymorphisms of *SOD2* in asthma. We show interactions on a multiplicative level (MEHHP residuals × *SOD2*) and interaction on an additive level (stratifying genetic effects by the limit of quantification of MEHHP). Although the multiplicative interaction term is statistically significant, the additive interaction (OR = 2.40 for the TC, and OR = 8.08 for the TT *SOD2* genotype) provides a better idea of the public health importance with 16.6% of the children being at risk with the TT and 43.3% having the TC genotype (Table 4). However, since *SOD2* and *GSTP1* are associated (Table S4), we cannot exclude that also *GSTP1* may also have an effect, which may only be detected in a larger cohort or when investigating a three-factor *SOD2* × *GSTP1* × phthalate interactions. Oxidative-stress-dependent pulmonary inflammation and tissue damage has been suggested to play a role in the pathogenesis of asthma [39]. In patients with asthma, the respiratory epithelium is considered to be deficient in its ability to neutralize oxidant attack through a decrease in antioxidant enzymes such as superoxide dismutase and glutathione peroxidase [28]. The *SOD2* gene variant was also found to be associated with childhood asthma [29,40]. Although the functional significance of these SNPs is not known and it is not known whether any functional polymorphism is in linkage disequilibrium with them, we found that individuals with risk alleles in the *SOD2* gene are more susceptible to phthalate exposure and that this sensitivity may be associated with phthalate related asthma. Further cohort studies are warranted to collaborate our genetic findings to characterize functional role of these markers in disease process.

### 4.4. The Combined Effect of Phthalate Metabolite Levels and Genotypes on Asthma

Phthalate is considered to induce oxidative stress by disrupting the activities of antioxidant enzymes [41]. And it has been reported that variations in *SOD2*, *GST*, and *EPHX1* genes contribute to diisocyanate susceptibility [16]. Then, oxidative stress may lead to airway inflammation and decrease pulmonary function as reported in rats exposed to pollutant mixtures [42]. A case-control study demonstrated that genetic variants of antioxidant defense genes, *SOD2*, *GST*, and *EPHX1*, were associated with increased susceptibility to diisocyanate-induced asthma [16]. In support of our findings, Park et al. found that *SOD2* polymorphisms modified the inverse association of urinary phthalate metabolites levels and pulmonary function in elderly Koreans [19]. Since oxidative stress is an early event in the pathway related to chemical-induced respiratory damage, genetic variability within genes coding for antioxidant defense systems might influence the ultimate expression of phthalate-induced asthma.

There are some limitations to this study. First, a case-control study can show associations but do not allow to assess the time-order. Second, asthma was diagnosed at 3 years of age, which might introduce misclassification. Though most clinicians agree that diagnosis before age 5 is very problematic, the airways have grown and widened by about 3 years of age, and the infant/toddler wheezing often stops. However, cases with asthma were further confirmed by pediatric allergists based on new criteria for the diagnosis and management of asthma in children under 5 years of age, see the GINA Guidelines 2015 [21]. Third, our study was limited by a focused use of a candidate gene approach. Traditional candidate gene approach is restricted to prior knowledge about functional aspects of possible candidates, which results in an information bottleneck. However, instead of only one candidate gene, we selected various candidate genes in one oxidative pathway for screening and validation. This approach adds biological plausibility to our study and was more cost-effective than the genome-wide approach. Fourth, our measurements are based on a single early morning urine sample in all children only. Previous studies have reported intra- and inter- variability in the concentrations of phthalate metabolites in a given subject’s urine samples [43,44]. However, spot urine samples and 24-h urine samples produced comparable estimates of daily DEHP intake [45]. Even though phthalates have relatively short half-lives, continuous daily exposure leads to an exposure scenario that is similar to persistent and bioaccumulative compounds [46]. If measurement error did occur, it tended to be toward the null and the effect of exposure was likely to be underestimated. Fifth, since the genotypes of *GSTP1* and *SOD2* are associated, we cannot exclude that *GSTP1* may also play a role, since our sample size is not large enough to investigate a gene × gene × phthalate interaction. Finally, there was no replication of this interaction in a separate cohort. Since these are novel preliminary findings and there are no similar studies at present, we tried to search for other cohorts with phthalate data to replicate our findings. Recently, a partial replication was conducted in a Korean cohort that also found an interaction between phthalate and *SOD2* for Forced Expiratory Volume/Forced Vital Capacity (FEV1/FVC) and Forced Expiratory Flow (FEF) 25–75 [19].

Using direct measurement of the exposure by objective quantitative biomarker for phthalate metabolites and of the outcome by physician diagnoses add to strength of our study. Assessing phthalate exposure by urine phthalate metabolites can reduce the recall bias of exposure assessment from questionnaires. Moreover, urine phthalate metabolites were analyzed by UPLC-MS/MS (Waters, Milford, MA, USA) with good validity, providing a reliable assessment of phthalate metabolites. In addition, we also adjusted for potential confounders such as urine creatinine, age, gender, maternal history of atopy, maternal education, and environmental tobacco smoke exposure. The combination of a well-designed epidemiological asthma study with a mechanistic approach addressing genetic variations in phthalate detoxification and oxidative stress provides novel insight into the research of the asthma and phthalate association.

## 5. Conclusions

Results of this case-control study suggests that phthalate exposure and *SOD2* rs5746136 variants were significantly associated with asthma. In addition, genetic variants of the *SOD2* gene might modify the association of phthalate exposure with asthma. Two future questions emerge due to these findings: First, is the expression of genes within the oxidative defense system triggered by phthalates and then results in a faster metabolization of phthalates? If so, does an increased activity of these genes then pose a higher risk for asthma? Second, would treatment with anti-oxidants protect against the elevated genetic risks for asthma by diminishing the oxidative defense system that may be triggered by phthalates? If so, preventive measures using anti-oxidants may avoid the effect of phthalate exposure in susceptible children. 

## Figures and Tables

**Table 1:** Characteristics of the study population.

Characteristic	Total	Cases	Controls	*p*-Value
Total number	453	126	327	
Mother				
Maternal age (years old)				
Mean ± SD	29.39 ± 4.35	29.61 ± 4.08	29.31 ± 4.45	0.559
Maternal education (%)				
High school and below	268 (59.2)	72 (57.1)	196 (59.9)	0.764
College and above	120 (26.5)	34 (27.0)	86 (26.3)	
Maternal history of atopy (%)				
Yes	126 (27.8)	46 (36.5)	80 (24.5)	0.002 *
Children				
Male (%)	261 (57.6)	76 (60.3)	185 (56.6)	0.470
Birth weight (gm)				
Mean ± SD	3119.33 ± 442.07	3075.94 (381.79)	3135.01 (461.56)	0.255
Premature birth (%)				
<37 weeks	35 (7.7)	9 (7.1)	26 (8.0)	0.993
Environmental factors				
Breast feeding (%)				
Yes	292 (64.5)	75 (59.5)	217 (66.4)	0.419
Older siblings (%)				
≥2	67 (14.8)	16 (12.7)	51 (15.6)	0.638
Pet raising (%)				
Yes	70 (15.5)	21 (16.7)	49 (15.0)	0.514
ETS exposure (%)				
Yes	186 (41.1)	59 (46.8)	127 (38.8)	0.025 *
Family income per year (U.S. dollars) (%)				
<20,000	110 (31.3)	25 (29.4)	85 (32.0)	0.808
20,000–50,000	213 (60.7)	52 (61.2)	161 (60.5)	
>50,000	28 (8.0)	8 (9.4)	20 (7.5)	

Abbreviations: ETS, environmental tobacco smoke; Some numbers do not add up to total *n* because of missing values; * *p* < 0.05.

**Table 2:** The association of urine phthalate metabolite levels and asthma (both measured at 3 years of age).

Asthma	Phthalate Metabolite Levels	*p*-Value	Bonferroni
Asthma Adjusted OR (95% CI) ^a^	LnMEP	0.803	1.000
	1.02 (0.85–1.24)		
Asthma Adjusted OR (95% CI) ^a^	LnMBP	0.678	1.000
	1.04 (0.87–1.25)		
Asthma Adjusted OR (95% CI) ^a^	LnMBzP	0.744	1.000
	1.04 (0.81–1.33)		
Asthma Adjusted OR (95% CI) ^a^	LnMEHHP	0.002 *	0.008 *
	1.33 (1.11–1.60)		

Abbreviations: OR, odds ratio; CI, confidence interval; * *p*<0.05; **^a^** Adjusted for urine creatinine, age, gender, maternal history of atopy, maternal education, and environmental tobacco smoke exposure; MEP: monoethyl phthalate; MBP: monobutyl phthalate; MBzP: monobenzyl phthalate; MEHHP: mono(2-ethyl-5-hydroxyhexyl)phthalate.

**Table 3:** The association of the genotypes of oxidative stress genes with asthma.

Genes (SNP)	Cases *n* (%)	Control *n* (%)	OR (95% CI)	Adjusted OR (95% CI) ^a^	Overall *p*-Value	Bonferroni *p*-Value	FDR
*GSTM1* (*n* = 453)	126	327			0.115	1.000	0.302
Present	68 (54.0)	203 (62.1)	0.72 (0.47–1.09)	0.77 (0.47–1.25)			
Null	58 (46.0)	124 (37.9)	1 (reference)	1 (reference)			
*GSTP1* (rs1695) (*n* = 452)	126	326			0.057	0.513	0.257
AA	87 (69.0)	205 (62.9)	2.61 (1.13–6.02) *	3.00 (1.23–7.33) *			
AG	32 (25.4)	78 (23.9)	2.52 (1.03–6.19) *	2.93 (1.13–7.59) *			
GG	7 (5.6)	43 (13.2)	1 (reference)	1 (reference)			
*SOD2* (rs5746136) (*n* = 453)	126	327			0.001 *	0.009 *	0.009 *
TT	30 (23.8)	45 (13.8)	2.70 (1.50–4.87) *	2.78 (1.54–5.02) *			
TC	60 (47.6)	136 (41.6)	1.80 (1.12–2.90) *	1.79 (1.12–2.89) *			
CC	36 (28.6)	146 (44.6)	1 (reference)	1 (reference)			
*SOD2* (rs4880) (*n* = 451)	126	325			0.235	1.000	0.302
GG	5 (4.0)	22 (6.8)	0.55 (0.20–1.50)	0.72 (0.19–2.66)			
AG	28 (22.2)	78 (24.0)	0.88 (0.54–1.44)	0.71 (0.38–1.32)			
AA	93 (73.8)	225 (69.2)	1 (reference)	1 (reference)			
*CAT* (rs769218) (*n* = 450)	126	324			0.218	1.000	0.302
AA	22 (17.5)	73 (22.5)	0.68 (0.37–1.24)	0.97 (0.47–1.98)			
AG	61 (48.4)	154 (47.5)	0.90 (0.56–1.43)	1.06 (0.59–1.89)			
GG	43 (34.1)	97 (29.9)	1 (reference)	1 (reference)			
*MPO* (rs2071409) (*n* = 446)	125	321			0.962	1.000	0.962
GG	6 (4.8)	11 (3.4)	1.37 (0.49–3.80)	0.71 (0.14–3.54)			
GT	20 (16.0)	61 (19.0)	0.82 (0.47–1.43)	0.61 (0.29–1.27)			
TT	99 (79.2)	249 (77.6)	1 (reference)	1 (reference)			
*EPHX1* (rs1051740) (*n* = 453)	126	327			0.321	1.000	0.361
CC	23 (18.3)	79 (24.2)	0.71 (0.39–1.29)	0.54 (0.26–1.13)			
TC	64 (50.8)	152 (46.5)	1.03 (0.64–1.65)	0.82 (0.45–1.47)			
TT	39 (31.0)	96 (29.4)	1 (reference)	1 (reference)			
*EPHX1* (rs2740171) (*n* = 445)	126	319			0.179	1.000	0.302
AA	15 (11.9)	19 (6.0)	2.07 (1.01–4.25) *	2.07 (1.00–4.24) *			
AC	18 (14.3)	56 (17.6)	0.84 (0.47–1.51)	0.84 (0.47–1.51)			
CC	93 (73.8)	244 (76.5)	1 (reference)	1 (reference)			

Abbreviations: OR, odds ratio; CI, confidence interval; SNP, single nucleotide polymorphism; FDR, false discovery rate; * *p* < 0.05; **^a^** Adjusted for age, gender, maternal history of atopy, maternal education, and environmental tobacco smoke exposure.

**Table 4:** The association of geometric means (s.e.) of urine phthalate metabolite levels (ng/mL) and different genotypes.

Genes (SNP)	*n* (%)	GM (s.e.) MEP	GM (s.e.) MBP	GM (s.e.) MBzP	GM (s.e.) MEHHP
*GSTM1* (*n* = 453)					
Present	271 (59.8)	15.57 (4.02)	4.48 (3.68)	0.60 (2.70)	5.37 (3.21)
Null	182 (40.2)	13.69 (3.69)	4.24 (3.53)	0.61 (2.65)	6.35 (2.86)
*GSTP1* (rs1695) (*n* = 452)					0.011 ^a^
AA	292 (64.6)	13.81 (3.78)	4.44 (3.74)	0.64 (2.64)	6.95 (3.12) *
AG	110 (24.3)	15.00 (3.43)	4.40 (3.31)	0.62 (2.75)	5.07 (2.66)
GG	50 (11.1)	17.52 (5.02)	3.70 (3.38)	0.50 (2.24)	3.35 (2.81) *
*SOD2* (rs5746136) (*n* = 453)					0.002 ^a^
TT	75 (16.6)	13.07 (3.43)	4.96 (3.99)	0.66 (3.21)	7.76 (3.67) *
TC	196 (43.3)	15.46 (3.85)	4.37 (3.64)	0.62 (2.48)	6.19 (2.94)
CC	182 (40.2)	13.92 (3.97)	4.06 (3.38)	0.58 (2.65)	5.08 (2.76) *
*SOD2* (rs4880) (*n* = 451)					
GG	27 (6.0)	14.20 (2.71)	4.30 (3.96)	0.48 (1.81)	4.04 (2.91)
AG	106 (23.5)	14.67 (3.53)	4.74 (3.77)	0.63 (2.86)	7.01 (3.02)
AA	318 (70.5)	14.29 (4.03)	4.19 (3.51)	0.61 (2.68)	5.76 (3.00)
*CAT* (rs769218) (*n* = 450)					
AA	95 (21.1)	17.52 (4.99)	4.60 (3.65)	0.52 (2.79)	5.37 (2.82)
AG	215 (47.8)	16.62 (4.06)	4.40 (3.46)	0.62 (2.74)	5.82 (2.90)
GG	140 (31.1)	12.14 (3.15)	4.07 (3.77)	0.66 (2.48)	6.60 (3.30)
*MPO* (rs2071409) (*n* = 446)					
GG	17 (3.8)	16.36 (3.52)	3.86 (5.56)	0.71 (3.21)	7.34 (4.70)
GT	81 (18.2)	16.40 (4.39)	5.20 (3.95)	0.66 (2.41)	6.17 (3.21)
TT	348 (78.0)	14.08 (3.69)	4.24 (3.44)	0.60 (2.72)	5.87 (2.91)
*EPHX1* (rs1051740) (*n* = 453)					
CC	102 (22.5)	15.56 (4.10)	4.77 (3.92)	0.65 (2.85)	6.11 (3.02)
TC	216 (47.7)	12.45 (3.39)	4.22 (3.49)	0.58 (2.48)	5.81 (2.96)
TT	135 (29.8)	17.21 (4.25)	4.21 (3.52)	0.62 (2.82)	6.02 (3.09)
*EPHX1* (rs2740171) (*n* = 445)					
AA	34 (7.6)	15.98 (3.17)	3.90 (4.63)	0.50 (1.98)	6.07 (2.95)
AC	74 (16.6)	13.46 (3.39)	5.17 (4.40)	0.65 (2.83)	6.16 (3.31)
CC	337 (75.7)	14.49 (4.02)	4.26 (3.36)	0.61 (2.71)	5.96 (2.96)

Abbreviations: SNP: single nucleotide polymorphism; GM (s.e.), geometric means (standard error); * *p* < 0.05; **^a^** *p*-value stands for the single significantly higher concentration compared to the other genotype.

**Table 5:** Additive interaction of *SOD2* genotypes identified by stratification by MEHHP (median and limit of detection level) and multiplicative interaction effect of the SOD2 gene and MEHHP residuals on asthma.

*SOD2* and MEHHP	Cases *n* (%)	Controls *n* (%)	OR (95% CI)	Adjusted OR (95% CI) ^a^
Model 1 (stratification) MEHHP (*n* = 453)	126	327		
MEHHP ≤ 4.14 ^b^ ng/mL (*n* = 310)	81	229		
*SOD2* (%)				
TT	14 (17.3)	30 (13.1)	1.77 (0.83–3.77)	1.77 (0.80–3.91)
TC	38 (46.9)	89 (38.9)	1.62 (0.93–2.83)	1.42 (0.77–2.62)
CC	29 (35.8)	110 (48.0)	1	1
MEHHP > 4.14 ng/mL (*n* = 143)	45	98		
*SOD2* (%)				
TT	16 (35.6)	15 (15.3)	5.49 (1.88–16.04) *	8.08 (2.03–32.14) *
TC	22 (48.9)	47 (48.0)	2.41 (0.93–6.26)	2.40 (0.69–8.40)
CC	7 (15.5)	36 (36.7)	1	1
Model 2 (interaction)	Genotype	β ^c^	OR (95% CI)	*p*-Value
*SOD2* (rs5746136)	TT	1.112	3.04 (1.61–5.73)	0.001 *
	TC	0.721	2.06 (1.22–3.48)	0.007 *
	CC		1	
Residuals of MEHHP		−0.047	0.95 (0.91–1.00)	0.062
*SOD2* (rs5746136) × Residuals of MEHHP	TT	0.055	1.06 (1.01–1.11)	0.035 *
	TC	0.057	1.06 (1.01–1.12)	0.031 *
	CC		1	

Model 1: = *SOD2* (rs5746136) for MEHHP < 4.14 ng/mL and/or ≥4.14 ng/mL; Model 2: = *SOD2* (rs5746136) + Residuals of MEHHP + *SOD2* (rs5746136) × Residuals of MEHHP; * *p* < 0.05; **^a^** Adjusted for urine creatinine, age, gender, maternal history of atopy, maternal education, and environmental tobacco smoke exposure; **^b^** 4.14 ng/mL is the limit of quantification and also the median. **^c^** The combined effect of *SOD2* TT with the residual of MEHHP can be estimated by: OR = exp(β_1_ ×*SOD2* TT + β_2_ × residual of MEHHP + β_3_ × (*SOD2* TT × residual of MEHHP)) = exp(1.112 ×*SOD2* TT − 0.047 × residual of MEHHP + 0.055 × (*SOD2* TT × residual of MEHHP)) = 3.04.

## Supplementary Materials

The following are available online at www.mdpi.com/1660-4601/14/2/162/s1, Table S1: The candidate SNP and TaqMan assay information (GRCh38.p7), Table S2: The distribution of urine phthalate metabolite levels (ng/mL); Table S3: The distribution of urine phthalate metabolite levels on asthma, Table S4: The association of *SOD2* and *GSTP1*.
