# Supplementary material for: Oxidative Stress-Related Genetic Variants May Modify Associations of Phthalate Exposures with Asthma
Source: Int J Environ Res Public Health. 2017 Feb 8;14(2):162. doi: 10.3390/ijerph14020162 (PMC5334716; doi:10.3390/ijerph14020162)
Supplement: Supplementary file 1 [file ijerph-14-00162-s001.pdf]

Supplementary Materials: Oxidative Stress-Related Genetic Variants May Modify Associations of Phthalate Exposures with Asthma

I-Jen Wang and Wilfried J. J. Karmaus

Table S1. The candidate SNP and TaqMan assay information (GRCh38.p7).

Gene	dbSNP	Location	TaqMan Assay ID	CEPH (CEU)	Yoruba (YRI)	Japanse (JPT)	Han Chinese (CHB)
SOD2	rs4880	chr6: 159692840	C__8709053_10	0.45 (C)	0.38 (C)	0.11 (C)	0.12 (C)
EPHX1	rs2740171	chr1: 225837827	C__16061129_10	0.22 (A)	0.39 (A)	0.19 (A)	0.07 (A)
MPO	rs2071409	chr17: 58270869	C__15868927_20	0.1 (C)	0.13 (C)	0.08 (C)	0.16 (C)
ARNT2	rs5000770	chr15: 80424141	C__3252950_10	0.32 (A)	0.08 (A)	0.42 (G)	0.37 (G)

SNP: single nucleotide polymorphisms; CEPH: Centre de'Etude du Polymorphism Humain.

Table S2. The distribution of urine phthalate metabolite levels (ng/mL).

Phthalate Metabolite	>LOD (%)	Minimum	Maximum	Geometric Means (s.e.)
MEP	83.9%	ND	1947.00	14.42 (3.82)
MBP	97.8%	ND	533.20	4.34 (3.59)
MBzP	97.6%	ND	156.40	0.61 (2.67)
MEHHP	91.7%	ND	664.40	5.94 (3.00)

LOD: Limit of detection; ND: Non-Detectable; The limit of detection for monoethyl phthalate (MEP), monobutyl phthalate (MBP), monobenzyl phthalate (MBzP), and mono(2-ethyl-5-hydroxyhexyl) phthalate (MEHHP) were 3.27, 0.95, 0.15, and 1.36 ng/mL.

Table S3. The distribution of urine phthalate metabolite levels on asthma.

Phthalate Metabolite	Cases GM (s.e.)	Controls GM (s.e.)	*p*-Value
**Total population (*n* = 453)**	126	327	
**MEP**	14.60 (3.89)	14.35 (3.80)	0.677
**MBP**	4.24 (3.88)	4.37 (3.49)	0.380
**MBzP**	0.63 (3.07)	0.58 (2.50)	0.942
**MEHHP**	6.99 (3.00)	5.57 (3.00)	0.020 *

Abbreviations: GM: geometric means; s.e.: standard error; * $p < 0.05$.

Table S4. The association of *SOD2* and *GSTP1*.

Gene(dbSNP)	Genotype	SOD2 (rs5746136)			Total
		TT *n* (%)	TC *n* (%)	CC *n* (%)	
	GG	2 (2.7)	15 (7.7)	33 (18.2)	50
GSTP1 (rs1695)	AG	15 (20.0)	52 (26.5)	43 (23.8)	110
	AA	58 (77.3)	129 (65.8)	105 (58.0)	292
	Total	75	196	181	452
		**Chi-square *p*-value:** 0.001 *			

* $p < 0.05$.

Exposure Monitoring

First mid-stream urine in the morning were collected and stored at −20 °C until analysis. Four phthalate metabolites (monoethyl phthalate (MEP), monobutyl phthalate (MBP), monobenzyl phthalate (MBzP), and mono(2-ethyl-5-hydroxyhexyl)phthalate (MEHHP)) representing the exposure to four commonly used phthalates (diethylphthalate (DEP), dibutyl phthalate (DBP), butyl benzyl phthalate (BBzP), and di(2-ethylhexyl) phthalate (DEHP)) were measured by ultra-performance liquid chromatography coupled with tandem mass spectrometry (UPLC-MS/MS). The samples passed through 96-well conditioned μ-Elution plates of Oasis WAX (Waters, Milford, MA, USA), washed

with 70% methanol, then the analytes were eluted with four times of 25 μL of 0.1% ammonium hydroxide in methanol. The eluent was dried and re-constituted with 100-μL methanol, and two micro-liters of the sample will be injected onto an Ascentis Express C18 column (50 × 2.1 mm, 2.7 μm, (Waters, Milford, MA, USA) with gradient elution using acetonitrile and 0.04% acetic $acid_{(aq)}$ as the mobile phase at a Waters ACQUITY UPLC, and the analytes were detected with a Quattro Premier XE triple-quadrupole mass spectrometer (Waters, Milford, MA, USA) using negative electrospray ionization (ESI-) using multiple reaction monitoring (MRM); two ion transitions of each analyted were acquired to fulfill the requirement of four identification points. The accuracy and precision of the analysis on real samples were based on two matrix spike samples (for accuracy and repeatability), sample duplicate (for repeatability), and sample spike (for accuracy). One blank and one quality control (QC) standard sample with the mixture of phthalate metabolites (100 ng/mL) were included in each batch of samples analyzed. Recovery of $^{13}C_{12}$-labeled internal-standard and native-standard of each phthalate metabolite in samples was higher than 85%. The phthalate metabolite levels of the blank samples were lower than twice the minimum detectable limit in each batch. The intra- and interassay coefficients of variation for the measurements of MEP, MBP, MBzP, and MEHHP in urine samples were 2% to 28%, 3% to 15%, 9% to 16%, and 10% to 20%. The calibration range of all phthalate metabolites was 0.2–1000 ppb. The R^2 was greater than 0.99 on each analyte. The limit of detection (LOD) for MEP, MBP, MBzP, MEHHP were 3.27, 0.95, 0.15, and 1.36 ng/mL. For concentrations below the detection limits, a value of half the lower limit of detection was assigned. The limit of quantitation (LOQ) for MEP, MBP, MBzP, MEHHP were 8.30, 1.77, 0.47, and 4.14 ng/mL. LOQ was determined based on signal-to-noise approach. Determination of the signal-to-noise ratio (10:1) was performed by comparing measured signals from samples with known low concentrations of analyte with those of blank samples and by establishing the minimum concentration at which the analyte can be reliably quantified. All results involved duplicate analysis. Regarding the procedure for avoiding contamination, the adsorbent was washed with twice of 0.1% NH_4OH in methanol and twice of Milli-Q water. All glassware was rinsed with acetone and methanol sequentially after washing and before the use. All adsorbent plate was disposed after the extraction. Each batch of samples contained a reagent blank, matrix blank, two matrix spike samples, sample duplicate, and sample spike. No contamination of analytes was identified. Urine creatinine levels were analyzed by enzymatic assay according to the manufacturers' instructions (Cayman Chemical, Ann Arbor, MI, USA) [1]. All statistical models were adjusted for urine creatinine levels.

References

1. Cayman Chemical Company. Creatinine (Urinary). 2012. Available online: https://www.caymanchem.com/pdfs/500701.pdf (accessed on 27 October 2016).

© 2017 by the authors; licensee MDPI, Basel, Switzerland. This article is an open access article distributed under the terms and conditions of the Creative Commons by Attribution (CC-BY) license (http://creativecommons.org/licenses/by/4.0/).
